# Sleep and Postpartum Psychosis: A Narrative Review of the Existing Literature

**DOI:** 10.3390/jcm12247550

**Published:** 2023-12-07

**Authors:** Camilla Carr, Daniela Borges, Katie Lewis, Jessica Heron, Sally Wilson, Matthew R. Broome, Ian Jones, Arianna Di Florio, Isabel Morales-Muñoz

**Affiliations:** 1 Institute for Mental Health, School of Psychology, University of Birmingham, Birmingham B15 2TT, UK; c.a.f.carr@bham.ac.uk (C.C.); d.s.a.borges@bham.ac.uk (D.B.);; 2 Coventry and Warwickshire Partnership NHS Trust, Coventry CV6 6NY, UK; 3 Division of Psychological Medicine and Clinical Neuroscience, Cardiff University, Cardiff CF24 4HQ, UK; 4 Action on Postpartum Psychosis, Swansea SA3 9BT, UK; 5 Birmingham Women’s and Children’s NHS Foundation Trust, Birmingham B4 6NH, UK

**Keywords:** postpartum psychosis, sleep, maternal mental health, narrative review

## Abstract

Sleep problems are extremely common during the postpartum period. The role of sleep in the development of postpartum psychosis (PP) is, however, still under-researched. This narrative review aims to (1) provide a summary of the existing evidence for the associations between sleep problems and PP, (2) discuss the relevant risk factors associated with sleep problems and PP, and (3) suggest future lines of research in this area. Some of the existing literature suggests an association between sleep problems, specifically insomnia, sleep loss and sleep disruption during pregnancy and postpartum, and PP, with the most relevant risk factors including history of bipolar disorder and time of delivery. However, it is still unclear whether the previously mentioned sleep problems are a symptom of, or a trigger for PP. Thus, further research is needed to identify the specific role of sleep problems in PP, using longitudinal designs and more objective measures of sleep. This will allow appropriate detection, intervention and support for women experiencing and/or at risk for PP.

## 1. Introduction

Postpartum psychosis (PP) is a mental health condition that occurs during the postpartum period, affecting an estimated 1–2 parous women in every 1000 deliveries [1]. PP is the term which refers to a severe form of psychotic mood disorder with a dramatic onset during the first four weeks after childbirth [2], and approximately 65% of these episodes occur in the first three days postpartum [3]. It commonly includes mania, psychotic depression, or mixed with features of both high and low mood. Additionally, it is common for women to show evidence of psychosis with delusions and hallucinations, and often demonstrate marked confusion or perplexity [4]. However, this condition is still highly understudied, with PP currently not being adequately dealt with in classification systems. Further, existing research on the factors associated with increasing risk of experiencing PP is limited and evidence for some potential risk factors is contradictory. For instance, although the evidence for bipolar disorder (BD) as a potential risk factor for PP is well-powered [5], evidence for other factors such as sleep problems pre- and perinatally is less clear. Examples of sleep problems in this context include short sleep duration, sleep fragmentation, parasomnias, insomnia, long sleep onset latency and/or sleep deprivation, among others. Although changes in sleep patterns can occur at any point in life, these sleep problems previously mentioned can increase the risk of experiencing PP in vulnerable mothers especially if they occur during pregnancy and postnatally. However, this is still an underexplored area of research.

Sleep research has received notable and increasing attention in recent years due to the emerging evidence supporting its role in many aspects of physical and mental health, including perinatal mental health conditions, such as PP. For instance, sleep loss during the postpartum period is very common, with sleep disruption occurring more frequently in the immediate postnatal period, coinciding with the onset of PP [6]. In addition, sleep disturbance, specifically a decrease in total sleep time and sleep efficacy, is most evident during the first month after childbirth, with primiparous women more likely to be affected by these sleep changes [7]. In relation to the specific sleep patterns that occur in mothers with PP, prolonged sleep latency, less total sleep time, more night awakenings, suppression of stage 4 sleep and a reduction of rapid eye movement (REM) sleep during the later stages of pregnancy and the postpartum period have been reported [8]. Further, the clinical link between sleep and PP has been known for centuries [9]. It is clear that sleep problems (e.g., insomnia) postnatally are symptoms of PP, but can also trigger the development of PP; therefore, understanding this distinction is extremely important for disentangling the role of sleep in PP. Insomnia has been identified as one of the most common symptoms of PP, with 42% to 100% of patients with PP experiencing any sleep disturbance during the postpartum period [10,11]. Moreover, insomnia has been found to precede the onset of PP symptoms [12]. Additionally, research has indicated sleep loss as a trigger or precedent of mania [13,14], which highlights the potential association between sleep and PP, given that the symptoms of mania and PP overlap and BD (of which mania is an episode) is also a potential precursor of PP [15]. Individuals with a diagnosis of BD are more likely to display irregular sleep-wake cycles and evening chronotypes [16], and have high polygenic liability to evening chronotype [17]. Moreover, irregularities in circadian rhythm are thought to contribute to mania and depression episodes [18]. As circadian rhythm disruption is common during the postpartum period and highly related to sleep problems and mood disorders [19], it is likely that it also plays a role in PP. The significance of sleep as a symptom and precursor of PP during the postpartum period supports the importance of focusing on sleep problems, including sleep loss, sleep disturbance and insomnia, as a potential risk factor for the development of PP.

To contribute to the existing literature on sleep and PP, this article provides a narrative review on the association between sleep problems (insomnia, sleep loss, sleep disturbance, etc.) and PP and relevant risk factors of these associations. More specifically, this narrative review aims to answer the following research questions:

- What is the association between sleep problems and PP, and when do these sleep problems usually occur?
- What are the relevant risk factors that may play a role in the association between sleep problems and PP?

## 2. What Is the Association between Sleep Problems and Postpartum Psychosis, and When Do These Sleep Problems Usually Occur?

Characteristics of the studies included are presented in Table 1. Firstly, a chart review conducted by Sharma et al. [12] used the data on duration of labour in 34 women and time of delivery in 42 women, admitted with a diagnosis of PP between the years 1990 and 2000, and found longer labours and night-time delivery to be associated with experience of PP (compared to controls who had no personal or family history of psychiatric illness). This association is thought to be the result of sleep deprivation due to giving birth during the night, which increases the likelihood of experiencing PP. However, no direct assessment of sleep was carried out, therefore it is difficult to conclude whether participants experienced any sleep loss as a result of night-time deliveries, in addition to sleep patterns before and during pregnancy. Additionally, the author mentioned that insomnia was found to be the most common symptom, and consistently preceded mood and psychotic symptoms, based on patient complaints and staff observations, which makes it difficult to distinguish whether sleep loss was due to a longer labour and night-time delivery, or insomnia.

Further, research conducted using a chart review, where electronic medical records were used to identify nine women with an ICD-10 diagnosis of PP and to determine SARS-CoV-2 infection status, has linked a decreased need for sleep to an increased risk of experiencing PP [25]. This research found that all women displayed profiles consistent with PP, including common symptoms such as a decreased need for sleep. Three women showed these symptoms of psychosis within a month after giving birth and after the SARS-CoV-2 infection. Furthermore, this research emphasizes the need for good sleep hygiene and ensuring that adequate sleep is being achieved in mothers during the perinatal period to decrease risk of PP.

In addition, two qualitative studies provided an in-depth insight into women’s experiences of PP and helped to understand the factors and symptoms that women believe to be an important aspect of this mental health condition. For instance, a recent qualitative study carried out in-depth semi-structured interviews with ten women and found that all participants reported sleep as a problem during their postpartum period [23]. Sleep problems presented by these women included not being able to sleep and being very awake/alert at night. However, these women noted that they had not realized that inadequate sleep was related to their symptoms until they recovered from PP. Furthermore, a study conducted using semi-structured interviews with nine registered nurses from hospital psychiatric departments in Sweden found, by using content analysis, that insomnia during the postpartum period (e.g., no sleep for a few nights or generally sleeping very little), was a symptom of PP [21]. However, this symptom was only identified by a minority of nurses (four out of nine), therefore it cannot be concluded that insomnia is a clear symptom of PP that occurs in every case. This may be as a result of the nurses having limited experience providing care for women with this illness, so they are not able to identify sleep problems as a symptom. Additionally, an element of bias may have been present as the nurses were reporting on patients with PP, who they were providing care for, therefore had likely established a rapport.

One longitudinal study does not support the link between sleep and psychosis both in pre- and postpartum periods. For instance, this study conducted by Mannion and Slade [24] in 66 participants measured sleep duration and sleep quality via a questionnaire (Pittsburgh Sleep Quality Index; PSQI) during pregnancy (28 week gestation) and the postnatal period (average 12.63 days after childbirth), and found that a large portion of the general population sample encountered subthreshold psychotic symptoms (e.g., delusion-like thoughts and perceptual aberrances, leading to distress and impairment) pre- and post-partum, despite not having a clinical diagnosis of psychosis. However, the authors found that participants did not significantly differ from puerperal populations outside of the study in terms of quality of sleep, suggesting that sleep may not be associated with experience of psychotic symptoms during the postpartum period. However, the sample was relatively biased in terms of ethnicity (i.e., 94% of white origin) compared to the general maternity population, which makes it more difficult to draw valid conclusions. Additionally, the questionnaires used to measure psychotic symptoms (i.e., Peters Delusions Inventory; PDI and Launay-Slade Hallucination Scale-Revised; LSHS-R) are not applicable to PP. For instance, this study found that 80% of their women had a psychotic symptom compared to 1 or 2 in every 1000 women who experience PP, thus their results cannot be generalized to the PP sample, which is the focus of this review.

## 3. What Risk Factors Are Associated with Sleep Problems and Postpartum Psychosis?

### 3.1. Bipolar Disorder

Within the existing literature, a few studies have noted the association between BD and sleep loss in relation to PP, suggesting that sleep disruption in the perinatal period increases risk of experiencing PP, particularly in women with a prior diagnosis of BD [3,14]. Given the clinical and mechanistic overlap between BD and PP [26,27], it is possible that the factors contributing to the association between BD and sleep loss are similar to those contributing to the association between PP and sleep (e.g., sleep loss, disturbance, etc.). A study by Lewis et al. [14] interviewed 870 parous women who met the DSM-IV criteria for BD, as women with BD are at an increased risk of affective psychoses after childbirth. The authors assessed whether episodes of mania or depression were triggered by sleep loss and found that 25.3% (*n* = 220) women reported sleep loss as a trigger of their episodes. In addition, it was discovered that women reporting sleep loss as a trigger had over double the odds of experiencing PP compared to women who did not report sleep loss. However, this study did not mention details on a comparison between women with BD who experienced PP and women with BD who gave birth but did not experience PP. Additionally, no objective data on sleep disruption during the postpartum period was included in this study, therefore it is difficult to conclude when the sleep loss occurred and the strength of sleep loss as a factor increasing risk of PP.

Furthermore, in a study on 127 women who experienced an episode of PP in the context of a bipolar illness, a quarter of participants reported not being able to sleep (23%) and not needing sleep (25%) as initial symptoms related to their illness [3]. However, the authors do not report the combined total for sleep-related symptoms in these women, therefore we cannot comment on whether these groups of women overlapped in reporting both symptoms. In addition, sleep and circadian rhythm disruption do increase the risk of mood disorder relapse in BD in women independent of childbirth [28]. However, it is still unclear whether the risk of relapse is greater for women who experience sleep disturbance associated with giving birth or at other times.

Finally, a smaller study comparing 27 women with a history of BD or PP to 17 controls and measuring sleep using sleep diaries and the Stanford Sleepiness Scale during pregnancy and in the postpartum period, did not find significant differences in sleep/wake activity between cases and controls [20]. Therefore, this provides evidence that sleep patterns between women with a history of BD or PP and controls are similar, thus may not play an important role in risk for experiencing PP. However, the sample size for this study was particularly small, meaning it may not have been well-powered enough to detect differences in sleep/wake activity between the groups. For future research, it would be very relevant to compare sleep patterns such as sleep/wake activity in women with BD who do or do not experience an episode of PP, in order to establish sleep disruption as a specific trigger.

### 3.2. Time of Delivery

The time of day that women give birth may be particularly important in the likelihood of experiencing PP, as it can be a marker of sleep disruption, specifically sleep deprivation. As mentioned above, Sharma et al. [12] conducted a chart review using data from women admitted with a diagnosis of PP between the years 1990 and 2000, and found an association between longer labours and night-time deliveries and development of PP (compared to controls), suggesting that the sleep deprivation caused as a result of giving birth during the night increased the likelihood of experiencing PP.

Additionally, a qualitative study by Engqvist and Nilsson [22], using open-ended interviews to gain an insight into experiences of PP in thirteen women, found that women encountered a number of unexpected and uncontrolled sleep problems after childbirth, including difficulties initiating sleep and sleep anxiety related to their child. Participants reported that even though they were exhausted, they could not fall asleep, and were consistently woken up by nurses to breastfeed the baby. These symptoms were found to coincide with childbirth during the night, providing support for previous research by Sharma et al. [12] suggesting night-time delivery and loss of sleep as precedents for PP.

## 4. Summary

This narrative review has presented the most relevant literature up to date investigating the associations between sleep and PP and other relevant risk factors. To summarize, there is still conflicting evidence regarding the specific role of sleep problems as a risk factor for PP. Although some of the existing research has found that insomnia, sleep loss and sleep disturbance postnatally increase the risk of experiencing PP, especially in those with a history of BD, the current evidence is not well-powered enough to make this conclusion. Consequently, the association between sleep and PP still remains unanswered, and thus further (and better designed) research is needed to provide a clearer answer for the role of sleep in risk for PP. Additionally, night-time deliveries and longer labours have also been found to coincide with experience of PP, most likely as a result of sleep loss and deprivation. Women have also identified the importance that sleep plays in their experience of PP, as well as hospital staff caring for women admitted with PP. However, most studies have been retrospective and prospective studies underpowered. Therefore, further research needs to be conducted with women at-risk or experiencing PP to gain a deeper understanding about sleep and the relevant risk factors which may play a role in the association between sleep and PP.

Based on the existing literature, it is likely that sleep problems (insomnia, sleep loss and sleep disruption) are associated with experiencing PP, either as a symptom or a risk factor. However, existing research up to date suggests that a history of mania triggered by sleep loss is associated with the onset of PP, which is noted in retrospective research suggesting that sleep loss triggering mania postnatally increases the risk of experiencing PP in women with a diagnosis of BD [14]. This would imply that sleep disruption after childbirth may increase risk of PP and could potentially trigger PP in women with increased vulnerability, such as those with a history of BD or previous experience of PP. Nevertheless, research has also shown that sleep is a symptom of PP [3,22]. Potentially, symptoms and triggers of PP may be characterised by different types of sleep problems. For example, sleep loss as a result of night-time delivery or anxiety from being a first-time mother may trigger a vulnerable individual to experience PP [12]. Further, PP may cause women to experience symptoms of insomnia or restlessness associated with mood changes [3]. Additionally, it is still unclear whether there is any specific sleep pattern associated with a higher risk of developing PP, compared to other sleep patterns. More knowledge about specific types of sleep problems would enable researchers to better understand the specific role of sleep in PP and help design early sleep intervention programs in mothers at high risk for PP. Furthermore, it may be useful for researchers to understand the role of sleep in other perinatal mental health conditions, such as postpartum depression, to build further on those interventions used to improve sleep problems, particularly as they may share similar characteristics and risk factors to PP.

### 4.1. Limitations of the Existing Literature and Current Review

First, the vast majority of research conducted on this topic has relied heavily on retrospective designs, where participants are asked to recall details about their experience of PP and relevant risk factors, which could consequently be vulnerable to recall bias. It also makes it extremely difficult to differentiate sleep problems as a symptom of PP or a trigger, due to the possibility of misremembering information. Second, the existing research conducted on sleep and PP has utilised different measures and designs (i.e., questionnaires, interviews), which are subjective in nature, and thus it may be difficult to draw valid conclusions as they may not measure the same sleep problem. Third, different studies have focused on different sleep variables (e.g., some research has measured sleep loss, whilst others have focused on sleep/wake activity or sleep quality), which does not allow for comparison of results from different studies. Therefore, the specific type of sleep problem should be taken into account when conducting research on risk factors, as some sleep problems might be more relevant than others. Based on the current existing research, insomnia, sleep loss and sleep deprivation are the sleep problems that have been most commonly explored within the context of sleep and PP, while other sleep problems (e.g., sleep fragmentation, nightmares, circadian rhythm disruptions and chronotype) have received less attention, and thus these are the sleep aspects that should be further investigated. Furthermore, there are likely to be different mechanisms involved for each sleep disorder which require the use of specific research approaches. Therefore, this should be considered when discussing the association between sleep and PP, as not all aspects of sleep will be a symptom or a risk factor. Fourth, this review included only publications written in English and excludes papers published in other languages. Finally, we were not able to include for this narrative review potentially relevant studies, such as Strouse et al. [29], as only the abstract of the study but not the full text was available. Therefore, the current narrative review is based only on those existing studies with the full text available.

### 4.2. Future Directions

First, and due to previous research suggesting an association between women diagnosed with BD and risk for PP, further research in women with BD should be implemented when conducting research on sleep and PP. Additionally, it is also extremely important for further research to investigate the role of circadian rhythm disruption in women with PP, as women with BD are characterised by irregularities in circadian rhythms which can increase risk of mood disorder relapse. However, it is important to differentiate between PP in the context of BD compared to PP alone. Typically, research has focused on PP in women with a history of BD; therefore, it is possible that PP manifests in more vulnerable women who experience sleep problems, rather than the result of perinatal sleep problems on their own. Identifying these individuals who are at an increased risk could enable early intervention and prevent vulnerable women from experiencing PP. Although, if the mechanisms are different between PP in the context of BD compared to PP alone, it may prove difficult to identify which individuals will experience PP as a result of sleep problems after childbirth.

Second, implementing sleep interventions which specifically target sleep during the perinatal period [8], educating women on good sleep hygiene given poor sleep quality during pregnancy predicts postpartum sleep and mood disturbances [30] and encouraging daytime deliveries in mothers at risk would be highly beneficial. However, the timing of labour and delivery can be highly unpredictable. Therefore, advising deliveries to occur during the day may not be a viable recommendation in most of the cases, but something that could be considered in those women at highest risk for PP. Furthermore, we still need to better understand the role of sleep as either a trigger or symptom of PP (or both) before putting these interventions into practice. For instance, Bergink et al. [2] trialled a peripartum prevention program to provide standardised evidence-based clinical care to women at high risk of relapsing during this period—which was designed taking into account the two strongest risk factors for PP (previous experience of PP and/or a history of BD). The program included recommendations for adequate sleep during the peripartum period alongside pharmacological interventions. This study found differences in clinical outcomes between women with a history of PP only compared to women with a diagnosis of BD significant, suggesting different routes of treatment should be taken depending on their history. Other potential suggestions for future sleep interventions include (1) encouraging postpartum patients with a diagnosis of BD to stay in the dark in the case of developing hypomania, given that bright lights can induce mania [31], (2) advise wake therapy in pregnant and PP patients, given an increase in total sleep time and improved mood occurring during the subsequent night in patients with postpartum depression [32,33], (3) using antipsychotics that target dopamine 2 receptors which may help to induce sleep, given the link between PP and dopamine receptor sensitivity [34], and (4) implementing strategies to help mothers sleep during the postpartum period, through encouraging postpartum women to seek help with breastfeeding early in the postpartum period (e.g., partner helping with bottle feeding).

Third, although previous research has found sleep to be a risk factor for PP, these conclusions are based on retrospective designs which may be prone to bias. Therefore, future research should focus more on longitudinal designs, which would enable researchers to conclude whether sleep is a symptom or a trigger for PP. Following individuals over a prolonged period of time would allow for the in-depth collection of data for a variety of sleep problems, which would further aid researchers in concluding whether sleep problems impact upon risk for PP.

Fourth, another future line of research is related to detailed investigation of specific sleep patterns as risk factors for PP. Although it has been hypothesised that changes to sleep and circadian rhythms during the perinatal period could be associated with increased risk of PP [26], evidence is lacking, and it is still unclear which specific sleep patterns are most relevant. For example, although existing evidence may suggest that insomnia precedes the onset of PP [12], no research to our knowledge has specifically investigated insomnia as a predictor of PP using a validated sleep measure. Thus, we cannot still conclude that insomnia is a predictor of PP. Additionally, existing research suggests that patients with postpartum depression show increased amounts of REM, and that shortened REM latency in pregnancy is found to be a predictor of postpartum depression [35]. Thus, it could be implied that since an association exists between postpartum depression and shortened REM latency, this altered sleep factor could also be a potential candidate for PP. Therefore, future research should focus on investigating a wider range of sleep and circadian factors, including sleep duration, sleep latency, chronotype, chronobiological disturbances, parasomnias or sleep quality, among others, using robust longitudinal designs to identify the specific type of sleep problems related to higher risk of experiencing PP. This will enable more targeted interventions and support for women at-risk of experiencing PP.

Fifth, future research would also benefit from using more objective and direct measures of sleep. Current research has mainly collected data on sleep using self-reported instruments (e.g., questionnaires and interviews), which are more subject to bias. Therefore, moving towards objectivity, for example through the use of actigraphy or polysomnography, would increase the reliability of findings and ensure sleep is being accurately measured. However, there are some problems with feasibility in relation to polysomnography; it typically only captures sleep for a few nights and involves participants being invited to a sleep lab which may prove difficult during the perinatal period, especially towards the end of pregnancy and in the postnatal period. Thus, a combination of both subjective and objective reports would be preferable, as both measures have limitations that need to be addressed. However, it is important to consider the mismatch between objective and subjective measures of sleep during pregnancy. For example, emotional distress (i.e., depressive and anxiety symptoms severity) during pregnancy is associated with subjective sleep disturbances but not with objective sleep disturbances [36]. Therefore, further research should focus on the potential differences between subjective and objective sleep in women with PP.

Sixth, many psychiatric disorders, which may be risk factors for PP (e.g., BD, depression and schizophrenia) are associated with sleep disorders, as previously reported [30]. Additionally, sensory deprivation is a potential contributing factor, given that chronic sleep deprivation combined with sensory deprivation is more likely to be associated with psychosis [37]. Therefore, these potential confounding variables in the association between sleep and PP should be taken into account for future research.

Finally, the majority of the mental health measures used in previous research (e.g., Edinburgh Postnatal Depression Scale and Mood Disorders Questionnaire) have not been validated for use in women experiencing PP. Although these measures have been used widely within this population, they were not designed for use within PP. Therefore, it is imperative that either current questionnaires are validated, or that new questionnaires are designed specifically to measure sleep and mental health in at-risk women for PP during and after pregnancy.

## 5. Conclusions

To conclude, although there is still conflicting evidence regarding the role of sleep in PP, some of the existing research suggests an association between sleep loss and sleep disturbance in the postpartum period and PP, with relevant factors including history of BD and time of delivery having a potential key role in these associations. However, sleep in PP is still widely understudied, with lack of consistency in the sleep problems being studied and methodology used. Therefore, future research needs to focus on using longitudinal designs to study the course of PP and to identify relevant risk factors associated with this disorder, as well as using a combination of subjective and objective measures of sleep. Understanding the role of sleep will enable better detection, early intervention and treatment for women at higher risk of experiencing PP.

## Figures and Tables

**Table 1:** Description of research on sleep and PP included in this narrative review (*n* = 8).

Original Studies	Study Design	N	Sample	Sleep Measure	Main Findings
Bilszta, Meyer & Buist (2010) [20]	Prospective	44 (27 with history of BD/PP, 17 controls)	Recruited via medical records, advertisements in support groups and mental health clinics	Sleep diary Stanford Sleepiness Scale (SSS)	No significant differences in sleep/wake activity between women with a history of BD/PP and controls
Engqvist et al. (2009) [21]	Descriptive investigation	9 registered nurses (8 females, 1 male)	Recruited voluntarily from hospital psychiatric departments in Sweden Age 39–60 years	Semi-structured interviews to help nurses describe symptoms of women who had experienced PP	Insomnia as a symptom of PP was identified by four nurses (e.g., going without sleep for a few nights or sleeping very little).
Engqvist & Nilsson (2013) [22]	Exploratory qualitative design, retrospective	13 women	Women considered themselves to be fully recovered from PP (time since experiencing PP ranged from 7 to 32 years) Age 39–60 years	Open-ended interviews to discuss their point of view and experience of PP	Women noted that they experienced unexpected and uncontrolled sleep problems after childbirth, such as difficulties initiating sleep, sleep anxiety, loss of sleep, etc. Symptoms coincide with childbirth during the night, which is consistent with previous research showing lack of sleep as a precedent for PP
Heron et al. (2008) [3]	Retrospective interview	127 women, episode of bipolar affective PP	Women had at least one episode of PP, recruited through APP	Women were asked to describe which symptoms they believe were first related to their illness—detailed description, impact on daily functioning, days of onset	29 women (23%) reported not being able to sleep as a symptom 32 women (25%) reported not needing sleep as a symptom
Jefferies, Schmied, Sheehan & Duff (2021) [23]	Qualitative interpretive research design using thematic analysis	10 women, aged between 28 and 35 when they had their first experience of PP	Invitation to participate was open to any woman in Australia who had experienced PP and recovered within the past ten years	In-depth semi-structured interviews to explore how women made meaning of their experiences of PP	Total number of PP episodes was 13 (*n* = 10). All 10 women reported sleep as an issue—after recovery, some realised that lack of sleep was a symptom of developing psychosis
Lewis et al. (2018) [14]	Retrospective	870 parous women	Bipolar Disorder Research Network (BDRN), met DSM-IV criteria for bipolar disorder, ≥18 years old	Interviews to assess whether episodes of mania or depression were triggered by sleep loss	25.3% (*n* = 220) reported sleep loss as a trigger for their mania episodes—these women were more likely to experience PP compared to those who did not report sleep loss as a trigger (2× the odds)
Mannion & Slade (2014) [24]	Longitudinal, prospective	101 women in their third trimester of pregnancy	98 participants were followed up after childbirth, of which 66 completed questionnaires postnatally	Questionnaires were sent within 7 days of childbirth, including Pittsburgh Sleep Quality Index (PSQI) to measure sleep duration and quality	Participants included in the study did not differ significantly from the general puerperal population in terms of quality of sleep (based on community norms for female samples)
Sharma, Smith & Khan (2004) [12]	Chart review, retrospective	Duration of labour = 34 women (17 psychosis, 17 controls) Time of delivery = 42 women (21 psychosis, 21 controls)	Women admitted with a diagnosis of PP, during 1990–2000 Controls had no personal or family history of psychiatric illness, same hospital as puerperal group	N/A	Sleep loss as a result of night-time deliveries is associated with the development of PP. Longer labour and night-time delivery can cause sleep deprivation which may trigger a mood episode

## Data Availability

Not applicable.
